# ABIN1 Inhibits Inflammation through Necroptosis-Dependent Pathway in Ulcerative Colitis

**DOI:** 10.1155/2022/9313559

**Published:** 2022-08-08

**Authors:** Jing Bao, Bin Ye, Yuhan Ren

**Affiliations:** Department of Gastroenterology, Shengzhou People's Hospital, Shaoxing, China

## Abstract

**Background:**

Ulcerative colitis (UC) is characterized by chronic, recurrent intestinal inflammation and intestinal epithelial injury including a wide range of epithelial cell death, ulcers, crypt abscesses, and the formation of fibrosis. The intestinal barrier dysfunction runs through the whole process of the occurrence and development of UC. A recent study revealed that an ubiquitin binding protein ABIN1 played a role in tissue homeostasis and autoimmunity diseases which involved in the anti-inflammatory response of intestinal epithelia cells. However, the roles of ABIN1 in ulcerative colitis pathogenesis remain unclear.

**Methods:**

The mRNA and protein expression level of ABIN1 and necroptosis-associated genes (RIPK1, RIPK3, and MLKL) were conducted to investigate the relationship between ABIN1 and necroptosis in clinical UC specimens. Subsequently, the dextran sodium sulfate (DSS)-induced mice colitis model was used to verify the ABIN1 function in vivo. Furthermore, we established ABIN1 gain and loss function assay in CACO-2 to confirm the mechanism in UC in vitro.

**Results:**

We found that ABIN1, RIPK1, RIPK3, and MLKL were upregulated in UC sample and DSS-induced colitis. Upon TNF-*α* stimulation in the intestinal epithelia cell line, overexpression of ABIN1 significantly inhibits necroptosis in the intestinal inflammation model along with the reduction expression of pro-inflammatory cytokines such as IL1B, IL6, IL8, and TNF-*α*. Blocking RIPK1 by Nec-1s *in vivo* and *in vitro* dramatically alleviated the colitis and cell death which shares the same phenotype with ABIN1 overexpression.

**Conclusion:**

Hence, the dysregulation of ABIN1 may relate to the uncontrolled necroptosis and inflammation in UC, and negatively regulate the occurrence and process of ulcerative colitis. ABIN1 activation may be considered a therapeutic strategy for UC.

## 1. Introduction

Inflammatory bowel disease (IBD) is mainly represented by Crohn's disease (CD) and ulcerative colitis (UC) [1]. Unlike Crohn's disease that could occur anywhere in total intestinal tract, UC primarily affects the colon and rectum, is characterized by chronic, recurrent intestinal inflammation and intestinal epithelial injury including a wide range of epithelial cell death, ulcers, crypt abscesses, and the formation of fibrosis. The etiology of UC is complex and diverse, which is related to monogenic/polygenic inheritance, microbial/environment, intestinal barrier, and intestinal immune system [2]. The intestinal barrier dysfunction runs through the whole process of the occurrence and development of UC [3]. And the intestinal barrier is mainly formed by the physical isolation of exogenous microorganisms and endogenous immune cells through the large amount of mucus secreted by intestinal cells and the tight intercellular junctions between intestinal epithelial cells [4]. Therefore, impaired intestinal mucosal barrier function may be an important factor in susceptibility to UC.

In the process of UC, it has been found that intestinal epithelial cells were involved in four kinds of death forms: apoptosis, pyroptosis, anoikis, and programmed necrosis (necroptosis) according to different cell subtypes [5]. Among them, necroptosis is most likely to cause inflammation, which is initially provoked by ligand biding to tumor necrosis factor receptors(TNFR) and then activates receptor interacting serine/threonine kinase 1(RIPK1) to recruit receptor interacting serine/threonine kinase 1(RIPK3) as downstream mediator and ultimately phosphorylate mixed lineage kinase domain-like(MLKL) protein results in membrane rupture, cell swelling, release of damage-associated molecular patterns (DAMPs) and leads to the development to various inflammatory diseases [6, 7].

Previous study shows that upon stimulation of TNF-*α*, an important proinflammatory cytokine involved in regulating various inflammatory diseases, RIPK1 acts as a critical regulator of necroptosis [8]. Activated RIPK1 has been indicated to be crucial for several inflammatory diseases including amyotrophic lateral sclerosis [9], multiple sclerosis [10], and IBD [11]. However, the kinase activity of RIPK1 is mediated by the post-translation modification such as phosphorylation and ubiquitination. According to the research of Slawomir et al., upon TNF-*α* stimulation, ABIN1, a ubiquitin binding protein, can suppress necroptosis by recruiting A20 to TNFR1 signaling complex (TNF-RSC) which mediates the deubiquitination of RIPK1 [12–14]. Furthermore, several genetic mutations of ABIN1 which lead to lower expression of ABIN1 are also dramatically related to some inflammatory diseases [15, 16]. Meanwhile, the survival of ABIN1-deficient mice can be prolonged through RIPK3 knockout or inactivation of RIPK1 to block necroptosis [12]. However, the contribution of the ABIN1 in ulcerative colitis pathogenesis remains unclear. Such evidence prompts that ABIN1 plays a critical role in suppressing RIPK1-mediated necroptosis in ulcerative colitis.

In this study, we investigate the expression level of ABIN1 and necroptosis key mediators in ulcerative colitis samples and dextran sodium sulfate-induced colitis models in mice. We found that ABIN1, RIPK1, RIPK3, and MLKL were upregulated in UC samples and DSS-induced colitis. Upon TNF-*α* stimulation in the intestinal epithelia cell line, overexpression of ABIN1 significantly inhibits necroptosis in the intestinal inflammation model along with the reduction expression of proinflammatory cytokines such as IL1B, IL8, and TNF-*α*. Blocking RIPK1 by Nec-1s *in vivo* (DSS-induced colitis in mice) and *in vitro* (TNF-*α* induced inflammation in the IEC cell line) dramatically alleviated the colitis and cell death which shares the same phenotype with ABIN1 overexpression. Hence, we hypothesized that the dysregulation of ABIN1 related to the uncontrolled necroptosis and inflammation in UC, and negatively regulates the occurrence and process of ulcerative colitis.

## 2. Method and Material

### 2.1. Patients' Specimens and Animal Treatment

For this study, normal (*n* = 10) and UC (*n* = 10) colon mucosal biopsies were obtained from healthy control and UC patients at Shengzhou People's Hospital from May 2014 to June 2017. The fresh UC specimens and normal mucosal were placed in liquid nitrogen for storage. All patients in this project have signed the informed consent and the study was reviewed and approved by the Ethics Committees of the Shengzhou People's Hospital. 6 to 8 week-old male C57BL/6 mice (20–24 g) were purchased from Charles River animal centre (Shanghai, China) and maintained in SPF facility. For the model of ulcerative colitis, mice were randomly divided into 4 groups (*n* = 6), vehicle group, DSS group, siABIN1+DSS group, and N1 + DSS + Nec-1s group. As described previously, mice were fed with 3.5% DSS (MP Biomedicals) for a week while the control group was given the same volume of distill water, as for siABIN1 + DSS group, mice were injected an additional 20 nm of siABIN1 (RIBOBIO, Guangzhou) every other day through tail vein. Meanwhile, the siABIN1 + DSS + Nec-1s group were added to daily drinking water based on the siABIN1 + DSS group. Each mouse was weighed and monitored for the appearance of blood and loose stool from day 1 to day 7. After a week, all mice were sacrificed, and then DAI score and the histopathological scores of mice were assessed as described in the previous work [17].

### 2.2. Cell Culture and Treatment

Human intestinal epithelia cell CACO-2 and 293T cell line were obtain from the American Type Culture Collection (ATCC). They were maintained in RPMI/1640 or high-glucose DMEM medium (Hyclone) supplemented with 10% fetal bovine serum (Gibco). Cells were cultured in a humidified incubator with 5% CO2 at 37°C. CACO-2 cell were treated with TNF-*α* (Sigma) and Nec-1s as described previously [17, 18]. As for RNA interference, cells were transfected with si-control and si-ABIN1, respectively, after premixing with Lipofectamine 3000 and incubated for 72 h.

si-ABIN1#1 GGTGCACACACCCTCGTATTC; si-ABIN1#2 GGAGCCTTCATAGGGACAGCC.

### 2.3. Extraction of RNA and Quantitative RT-PCR

Patients' mucosal, DSS-induced colon tissue and CACO-2 cell were applied for total RNA extraction. Total RNA was reverse transcribed into cDNA by TOYOBO ReverTra Ace kit (TOYOBO). Subsequently, 0.5 ug cDNA was subjected by using the SYBR system (Takara) following the manufacturer's instructions in an CFX (Bio-Rad) real-time PCR machine. The mRNA expression level was calculated by the 2^−ΔΔCt^ method. The final values were normalized to the mRNA expression of *β*-ACTIN. The primers of *β*-ACTIN, ABIN1, RIPK1, MLKL, IL1B, IL8, TNF-*α*, and RIPK3 used for qRT-PCR are shown in Table 1.

### 2.4. Western Blot Analysis

Total proteins were extracted from patents' specimens, DSS-induced colon tissue, and cell lines by 1% NP40 lysis. Protein concentration was detected by Braford Kit (Beyotime). In performing Western blot, 20 mg protein were separated on different concentrations of SDS-PAGE gel and by following the standard protocol of WB. After ECL exposure, the protein expression level was calculated by gray value through ImageJ.

### 2.5. Statistical Analysis

The results are shown as the mean ± SD. Differences in mean values between two groups were analyzed by two-tailed Student's *t* test. *P* values less than 0.05 were considered statistically significant, and the statistical analysis was conducted in GraphPad Prism6.

## 3. Results

### 3.1. ABIN1 is Markedly Increased in the UC Samples

**Figure 1:**
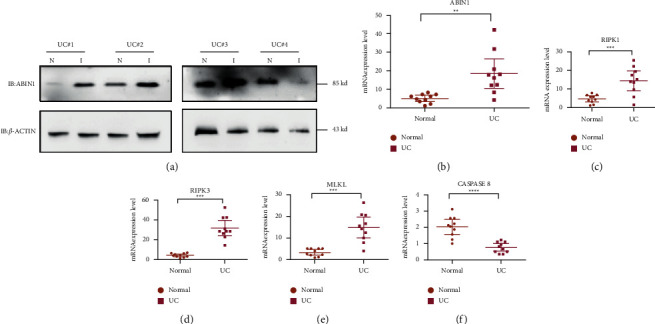
ABIN1 is markedly increased in the UC samples. (a) ABIN1 protein expression level was assessed in 4 pairs of UC patients; (b)–(f) the mRNA expression level of ABIN1, RIPK1, RIPK3, MLKL, and CASPASE8 were detected in ten pairs of UC patients' mucosal and adjacent normal tissues. Two-tailed student's *t*-test was performed to assess statistical significance ^*∗∗*^*P* < 0.01, ^*∗∗∗*^*P* < 0.001.

ABIN1 plays a role in tissue homeostasis and autoimmunity diseases which involved in the anti-inflammatory response of intestinal epithelia cells. So, we investigate the mRNA and protein expression level of ABIN1 in 10 pairs of UC samples. The results show that ABIN1 mRNA expression level was significantly upregulated with value *P* < 0.017 (Figure 1(a)) and 3 in 3 pairs of ABIN1 protein expression level in inflamed colon were significantly increased as well (Figure 1(b)).

As discussed in the previous study, ABIN1 coordinates with A20 and regulates the ubiquitination of core necroptosis mediator RIPK1. We investigate the necroptosis level by determining mRNA expression level of CASPASE8, RIPK1, RIPK3, and MLKL in UC specimens compared with these levels in the adjacent normal tissues. The results show a remarkable increase in RIPK1 (*P* < 0.009), RIPK3 (*P* < 0.001), and MLKL (*P* < 0.001) in mRNA expression level (Figures 1(c)–1(e)), and CASPASE8 was significantly downregulated in UC (Figure 1(f)) indicating ABIN1 involvement causing increased necroptosis level *in vivo*.

### 3.2. ABIN1 Inhibits Necroptosis and the Expression of Proinflammatory Cytokines in IEC

**Figure 2:**
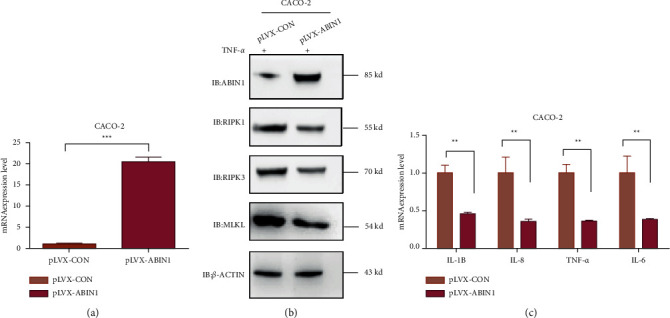
ABIN1 overexpression in IEC inhibits necroptosis and the expression of proinflammatory cytokines in IEC: (a) mRNA expression level overexpression verification in CACO-2 cells; (b) Necroptosis-associated proteins detection in CACO-2 ABIN1 overexpression stable upon TNF-*α* stimulation; and (c) mRNA expression level of proinflammatory cytokines in ABIN1 overexpression CACO-2 cells. Two-tailed Student's *t*-test was performed to assess statistical significance. ^*∗∗*^*P* < 0.01, ^*∗∗∗*^*P* < 0.001.

**Figure 3:**
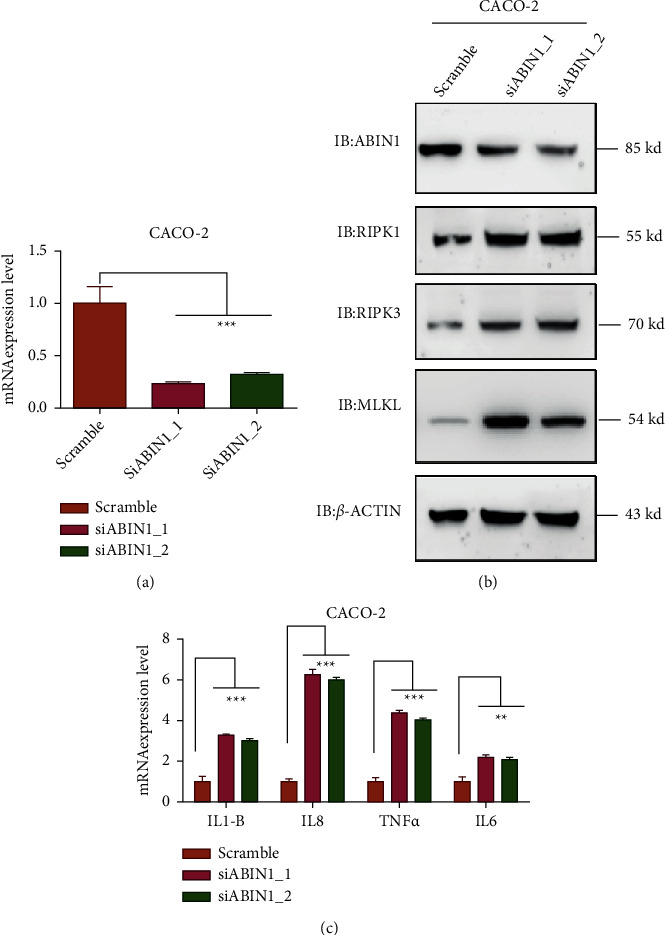
ABIN1 knockdown in IEC promotes necroptosis and the expression of proinflammatory cytokines in IEC. (a) mRNA expression level siRNA verification in CACO-2 cells; (b) Necroptosis-associated proteins detection in CACO-2 ABIN1 knock down stable upon TNF-*α* stimulation; and (c) ABIN1 knock down CACO-2 cells. Two-tailed Student's *t*-test was performed to assess statistical significance. ^*∗∗*^*P* < 0.01, ^*∗∗∗*^*P* < 0.001.

To unveil the function of ABIN1 in mediating necroptosis and intestinal inflammation, we constructed an ABIN1 ectopic expression stable in the intestinal epithelial cell line. As shown in (Figures 2(a) and 2(b)), we successfully established ANBIN1 overexpression in the CACO-2 cell line. Upon TNF-*α* stimulation in CACO-2 cells, overexpression of ABIN1 remarkably reduced the mRNA expression level of proinflammatory cytokines including TNF-*α* (Figure 2(c)), IL-6 (Figure 2(c)), IL1B (Figure 2(c)), and IL8 (Figure 2(c)). Besides the protein expression of necroptosis core components RIPK1, RIPK3, and MLKL were decreased as well (Figure 2(b)). To further verify the function of ABIN1 in IEC, we used siRNA of ABIN1 to block the expression in CACO-2 cells (Figure 3(a)). The results uncovered that knockdown ABIN1 leads to the accentuation of necroptosis through increasing the expression of RIPK1, RIPK3, and MLKL (Figure 3(b)). Meanwhile, the expression of TNF-*α*, IL-6, IL1B, and IL8 were upregulated under TNF-*α* stimulation *in vitro* (Figure 3(c)).

### 3.3. Nec-1s Mitigates the Necroptosis-Associated Protein Expression and Intestinal Damage in ABIN1 Deficiency DSS-Induced Mice

**Figure 4:**
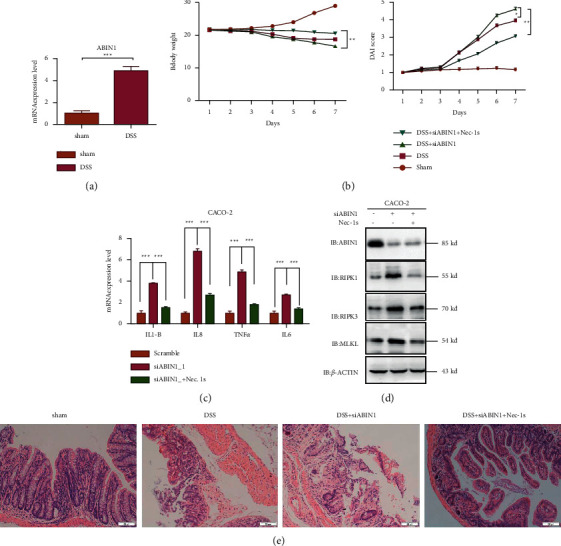
Nec-1s mitigate the necroptosis-associated protein expression and intestinal damage *in vivo and in vitro*. (a) mRNA expression level of ABIN1 in the DSS-induced colitis model; (b) the body weight and DAI index change in mice on combination treatment with DSS, siABIN1 and Nec-1s for 7 days; (c) mRNA expression level of proinflammatory cytokines under ABIN1 ablation and Nec-1s treatment in CACO-2; (d) necroptosis-associated proteins detection expression level under ABIN1 ablation and Nec-1s treatment in CACO-2; and (e) representative images of four groups' colonic tissue stained with hematoxylin and eosin (H&E; upper) are shown. The bar represents 50 *μ*m and two-tailed student's *t*-test was performed to assess statistical significance. ^*∗∗*^*P* < 0.01, ^*∗∗∗*^*P* < 0.001.

Nec-1s, a RIPK1 inhibitor which plays a potent role in anti-inflammatory, was subjected to investigate the relationship between ABIN1 and RIPK1 in the DSS-induced colitis model. Mice were treated with 2.5% DSS for 7 days to bring symptoms of UC, such as loose stool, diarrhea, and weight loss, and then, we maintained DSS-induced mice with a daily treatment of si-ABIN1 or si-ABIN1 combined with Nec-1s treatment. We detected the mRNA expression level of ABIN1 in the DSS-induced colitis model (Figure 4(a)). The daily change of body weight and DAI was represented in Figure 4(a). The body weight of DSS + si-ABIN1 treatment group was significantly reduced from day 6 to day 7 compared to the DSS-treated group (DSS vs DSS + si-ABIN1, 18.52 ± 0.42 vs. 15 ± 0.32, *P* < 0.05); however, the body weight of the combination of DSS, si-ABIN1, and Nec-1s treatment group was conversely recovered when compared to DSS + si-ABIN1 group (DSS + si-ABIN1vs DSS + ABIN1 + Nec-1s, 15.25 ± 0.36 vs. 20.29 ± 0.22, *P* < 0.01). As for DAI comparison (Figure 4(b)), the similar result had shown combining Nec-1s mitigates the DAI, which is aggravated by ABIN1 deficiency. (DSS + si-ABIN1 vs. DSS + ABIN1+Nec-1s, 4.6 ± 0.38 vs 3.28 ± 0.21., *P* < 0.01).

Next, HE staining of the colon was conducted to evaluate the histological changes. DSS treatment caused muscle thickening, crypt damage, and lymphocyte infiltration and si-ABIN1 aggravates the intestinal injuries; however, adding the Nec-1s, inhibitor of RIPK1, greatly reduced these symptoms (Figure 4(c)). To further testify the relationship of ABIN1- and RIPK1-related necroptosis *in vitro*, we exposed colonic epithelial cell (Caco2) to Nec-1s upon TNF-*α* stimulation with or without the ablation of ABIN1. The results showed that Nec-1s could restore the increase of proinflammatory cytokines induced by ABIN1 ablation. And the upregulation of necroptosis-associated protein by ABIN1 ablation is also inhibited by the treatment of Nec-1s (Figure 4(d)).

## 4. Discussion

Intestinal barrier injury is the main symptom of UC which is induced by the abnormal cell death and proliferation in IEC. Cell death can be divided into four forms such as apoptosis, pyroptosis, anoikis, and necroptosis [5]. Unlike other forms of death forms, necroptosis was mainly induced in the TNF-*α*-mediating pathway which led to imbalance in cell death and proliferation of IEC. In necroptosis, RIPK1 is the core mediator which was initially discovered by screening for proteins that bind to the death receptor Fas [19]. Subsequently, research studies confirmed that RIPK1 was involved in TNF-*α* signaling as a skeleton protein to activate the NF-*κ*B pathway [20]. Then, in 2000, Holler et al. [8] found that RIPK1 kinases were related in cell non-caspase-dependent necrosis. In 2005, Degterev et al. [21] formally named RIPK1-dependent cell necrosis as programmed necrosis. Thus, RIPK1 is a dual-functional protein: one function depends on its protein skeleton and the other on its kinase activity. The functional diversity of RIPK1 is also reflected in transgenic mice. Mice with RIPK1 protein deletion can only survive for 3 days [22], but mice with RIPK1 kinase activity deletion can survive normally [23]. In addition, it has also been reported in recent years that RIPK1 is related to human inflammatory diseases [24]. The deletion mutation of RIPK1 gene can lead to severe immune deficiency and other diseases, while another missense mutation of RIPK1 gene can lead to inflammatory diseases [25]. Knockout of FADD based on RIPK1 IEC-KO saved premature death and weight loss in mice, suggesting that loss of RIPK1 gene in IECs leads to FADD-dependent apoptosis and intestinal inflammation [26].

In terms of mechanism research, the connection between RIPK1 and IBD was also reported in 2014. Dannappel et al. [27] found that mice with IECs conditional deletion of RIPK1 gene (RIPK1 IEC-KO) would die within 4 weeks after birth, accompanied by inflammatory infiltration of intestinal tissue. The results were similar to those of RIPK1 IEC-KO mice, indicating that RIPK1 gene not only maintains the homeostasis of newborn mice but also plays a crucial role in adult mice IECs. Belgian Takahashi et al. also reported similar experimental results [28]. They used RIPK1 IEC-KO mice to study and found that Caspase 8 knockout could save the phenotype of RIPK1 IEC-KO mice [29]. Both studies demonstrated that the importance of RIPK1 in the regulation of FADD and Caspase 8 genes in intestinal epidermal cells was consistent with the key regulatory position of RIPK1 protein in the TNF-*α* signaling pathway [27].

As we know, currently the medical UC therapies were based on traditional 5-aminosalicylate and corticosteroids and new classes of biologic drugs mainly refer to tumor necrosis factor (TNF) antagonists including infliximab, adalimumab, and certuzumab [30]. Compelling evidence shows that anti-TNF therapy remains one of the most effective ways for treating UC. Furthermore, previous studies shown that the TNF-*α* signaling pathway was closely related to necroptosis in the absence of caspase 8 [8]. However, the relationship between necroptosis and UC remains largely obscure. In this study, we found that caspase 8 expression level in UC patients' mucosa remarkably decreased compared to healthy patients' mucosa along with the increasing expression of necroptosis-associated protein RIPK1, RIPK3, and MLKL. And the similar results were uncovered in the DSS-induced colitis model. Previous research showed that upon TNF-*α* stimulation, deficiency of caspase 8 leads to the inhibition of apoptosis and results in the upregulation of RIPK1-RIPK3-MLKL complex II-related necroptosis [31]. It implies that necroptosis plays a main role in the inflammation of UC in a TNF-*α*-mediating pathway.

ABIN1 is a polyubiquitin-binding protein known to bind linear ubiquitin chains on TNF-RSC and A20 to mediate the deubiquitination of RIPK1 to suppress necroptosis during development [32]. Besides, ABIN1 and A20 are linked to several inflammatory disorders [15, 16]. A20 protein is an anti-inflammatory molecule that inhibits NF-*κ*B inflammatory signaling and cell death downstream of TNF-*α*. Mutations in its coding genes have been confirmed to be associated with a variety of inflammatory diseases, such as CD and rheumatoid arthritis [33]. In 2018, it was reported that simultaneous conditional knockout of the A20 and ABIN1 genes of IECs led to the failure of mice to survive and the rapid death of IECs. In *in vitro* cell experiments, inhibition of RIPK1 kinase activity by Nec-1s could save the death of IECs cells [34]. Ricard et al. further studied the expression of A20 in IBD patients' tissues, and found that in the ileum and colon of CD patients, A20 is as highly expressed as TNF-*α*. Upon TNF-*α* stress, in A20-TG mice and enteroid of A20-TG, IECs cells began to undergo apoptosis, which was reversed by RIPK1 inhibitor, suggesting that A20 overexpressed intestinal cells which undergo RIPK1-dependent apoptosis by the action of inflammatory factors. In addition, its paralogous gene, ABIN3, shows negatively regulated necroptosis-induced intestinal inflammation by interacting with A20 and restricting the ubiquitination modification of necroptosis in inflammatory bowel disease [31]. So, we investigate the inner function of ABIN1 and RIPK1 in UC. In this study, we found ABIN1 expression level in UC patients' mucosa remarkably increased compared to health mucosa. To further understand the function of ABIN1 in UC, we established the ABIN1 overexpression and knock down in vitro model in CACO-2 cell. We found that overexpression ABIN1 in TNF-*α*-induced CACO-2 cell leads to the reduction expression of necroptosis core components such as RIPK1, RIPK3, and MLKs. Besides the expression of proinflammatory cytokines including TNF-*α*, IL-6, IL1B, and IL8 were decreased as well. We knocked down ABIN1 in CACO-2 cell which showed the converse results. And then, we blocked RIPK1 with Nec-1s in the ABIN1 deficiency model which also inhibited the increase of proinflammatory cytokines and necroptosis-associated proteins. Subsequently, we performed *in vivo* experiments to further investigate the role of ABIN1 in UC. We exposed DSS-induced colitis mice with siABIN1/Nec-1s treatment to assess the function of ABIN1 in the UC necroptosis model. Our results showed that with the deficiency of ABIN1, the inflammation that induced by DSS was aggravated, and Nec-1s can restore the effects.

Collectively, our study shows that ABIN1 alleviated UC inflammation through a TNF-stimulation necroptosis-dependent pathway. These results provide insights into UC etiology and suggest that ABIN1 activation may be considered a therapeutic target for treating UC.

## Figures and Tables

**Table 1:** The sequence of qRT-pcr primers.

qRT-PCR primer	Forward (5′–3′)	Reverse (5′–3′)
*β*-ACTIN	CACCATTGGCAATGAGCGGTTC	AGGTCTTTGCGGATGTCCACGT
RIPK1	TATCCCAGTGCCTGAGACCAAC	GTAGGCTCCAATCTGAATGCCAG
RIPK3	GCTACGATGTGGCGGTCAAGAT	TTGGTCCCAGTTCACCTTCTCG
MLKL	TCACACTTGGCAAGCGCATGGT	GTAGCCTTGAGTTACCAGGAAGT
IL1B	CCACAGACCTTCCAGGAGAATG	GTGCAGTTCAGTGATCGTACAGG
IL6	AGACAGCCACTCACCTCTTCAG	TTCTGCCAGTGCCTCTTTGCTG
IL8	GAGAGTGATTGAGAGTGGACC	CACAACCCTCTGCACCCAGTTT
TNF-*α*	CTCTTCTGCCTGCTGCACTTTG	ATGGGCTACAGGCTTGTCACTC

## Data Availability

All the data can be acquired by reasonable request.
